# Supplementary material for: MutationalPatterns: the one stop shop for the analysis of mutational processes
Source: BMC Genomics. 2022 Feb 15;23:134. doi: 10.1186/s12864-022-08357-3 (PMC8845394; doi:10.1186/s12864-022-08357-3)
Supplement: Supplementary file 3 — Additional file 3: Additional tables containing extra data that can help the reader better understand the manuscript. Table S1. List of new features and bugfixes. Table S2. The potential damage of mutational signatures. Table S3. The genes used for the signature potential damage analysis. Table S4. Overview of the source of the replication timing data. [file 12864_2022_8357_MOESM3_ESM.docx]

**Table S1: List of new features and bugfixes**

| New features: |
| --- |
| Added support for Indel variants. Mutation matrixes and profile plots can be generated for these variants. Signature extraction and refitting is also possible. |
| Added support for DBS variants. Mutation matrixes and profile plots can be generated for these variants. Signature extraction and refitting is also possible. |
| Added support for MBS variants. Mutation matrixes and profile plots can be generated for these variants. Signature extraction and refitting is also possible. |
| The SNV profiles are now cleaner, because the DBS and MBS support means they are no longer classified as SNVs. |
| Added the get_mut_type function, which allows users to filter for a mutation type of interest. A shortcut to do this directly via read_vcfs_as_granges is also supported. |
| The mut_matrix, mut_matrix_stranded, mut_context and type_context functions now support a wider mutational context. |
| Mutational SNV profiles can be visualized as a heatmap with the plot_profile_heatmap function. This supports profiles that have a wider context. |
| Mutational SNV profiles can be visualized as a riverplot with the plot_river function. This supports profiles that have a wider context. |
| Added the fit_to_signatures_strict function. This function allows for more stringent signature refitting, which doesn't suffer from overfitting. |
| Added support for bootstrapped signature refitting. The fit_to_signatures_bootstrapped function can be used to determine the confidence of signature refits. The plot_bootstrapped_contribution function can be used to plot the results as a dotplot, jitter plot or bar graph. |
| Added support for region specific analyses. Mutations can now be split based on user defined regions with the split_muts_region function. This way regions can be treated as separate samples. Instead of treating regions as samples, they can also be treated as mutation features with the lengthen_mut_matrix function. These two new functions together with existing functions allow for the extraction of signatures that are region specific or that have different mutation contexts in different regions. Graphs to compare the spectra and profiles between different regions can be made, using the plot_spectrum_region and plot_profile_region functions. |
| Added support for lesion segregation analyses. The presence of lesion segregation can be calculated in multiple ways using the calculate_lesion_segregation function. Lesion segregation can also be visualized with the plot_lesion_segregation function. |
| mut_matrix and mut_matrix_stranded are now vectorized, which improves their runtime. |
| The potential damage that a signature can do can be determined by looking at the number of "stop gain", "mismatch", "synonymous" or "splice site" mutations it could cause in a user supplied set of genes. This is first done per mutational context with the context_potential_damage_analysis function. Its output can then be used with the signature_potential_damage_analysis function. |
| The COSMIC signatures have been updated to version 3.2 (Alexandrov, L.B. et al., 2020, Nature). Additionally, we added the SIGNAL and Sparse signatures (Andrea Degasperi et al., 2020, Nature Cancer; Daniele Ramazzotti et al., 2019, Bioarchive). Signatures can now be easily retreived with the get_known_signatures function. |
| The plot_spectrum function can now plot individual samples as points. |
| The plot_spectrum function can now use a 95% confidence interval (default), standard deviation or standard error of the mean for its error bars. Previously, only the standard deviation was supported. |
| Unit tests have been made for all functions, resulting in a test coverage of more than 90%. |
| The extract_signatures function now supports a variational bayes NMF algorithm from the ccfindR package. |
| The enrichment_depletion_test and strand_bias_test functions now calculate a fdr value in addition to a p-value. |
| The cutoff p-value or fdr-value for the enrichment_depletion_test and strand_bias_test functions can now be supplied by the user. Multiple cutoff values can be used, which will result in multiple significance stars. |
| Extracted signatures can now be renamed based on already existing signatures, using the rename_nmf_signatures function. |
| The cosine similarity between reconstructed profiles and the original can be plotted with the plot_original_vs_reconstructed function. |
| Signatures that are very similar can be merged with the merge_signatures function. |
| Tissue specific signature contributions can be converted to reference signatures using the convert_sigs_to_ref function. |
| Regions with different mutation densities can be determined using the bin_mutation_density function. This can be useful for region specific analyses. |
| Improved single sample support. Functions like mut_matrix and mut_matrix_stranded now work on a single GRanges object, instead of only on a GRangesList. |
| The plot_contribution_heatmap and plot_cosine_heatmap functions can now perform clustering on both their rows and their columns. A predetermined order can also be given for both the rows and the columns. |
| Multiple samples within a mutation matrix can now be pooled together using the pool_mut_mat function, to increase statistical power. |
| Tests are performed to check whether input GRanges objects match the supplied reference genome. Clear error messages are generated when this is not the case. |
| The plot_contribution function no longer requires a signature matrix as input, when working on signature refitting data. |
| Instead of supplying the name of a BSgenome object, as an argument to functions that require it, users can now also directly use the object itself. |
| Regions with a different mutational profile can be determined in an unsupervised way, using the determine_regional_similarity function. These regions can be visualized using the plot_regional_similarity function |
| Bugfixes: |
| A bug in the mut_strand function caused incorrect results for replication strand bias analyses. This is now fixed. |
| The binomial_test function is now properly two-sided. |
| The plot_rainfall function now sorts its input. This prevents incorrect results on unsorted input. |
| The mutations_from_vcf didn't work on empty GRanges objects. This is now fixed. |
| Removed messages about using certain variables as a "grouping variable". |
| Removed warnings about using alpha as a variable. |
| The read_vcfs_as_granges function no longer throws an error, when trying to read in an empty vcf file. |
| The alignment of angled x-axes on plots has been fixed. This issue was most notable for the heatmaps. |
| The read_vcfs_as_granges function no longer supplies a genome name to the internally used readVcf function. This fixed an issue caused by updated to the seqnames dependency. |
| When the seqlevelStyle can’t be changed by read_vcfs_as_granges or when the filtering of seqlevel groups doesn’t work, this is said in a clear error message. It’s now possible to not change the seqlevelstyle to circumvent these errors. |
| The defunct functions mutation_context, mutation_types, strand_from_vcf and explained_by_signatures have been removed. |
| The plot_rainfall function plots "chr1" as 1, to save space. This is now case-insesitive, so it will also work on "Chr1". |
| The plot_rainfall function now gives a clearer error when a GRangesList is used, instead of a GRanges object. |

**Table S2: The potential damage of mutational signatures**

| Mutation damage type | signature | ratio | ratio_by_flat_background_signature | Total number of potential mutations |
| --- | --- | --- | --- | --- |
| Stop_gain | SBS1 | 0.035157449 | 0.81 | 17.32 |
| Missense | SBS1 | 0.612255434 | 0.86 | 281.87 |
| Synonymous | SBS1 | 0.340418953 | 1.57 | 149.09 |
| splice_site | SBS1 | 0.012168163 | 0.40 | 5.98 |
| Stop_gain | SBS10a | 0.155552474 | 3.60 | 166.07 |
| Missense | SBS10a | 0.690129545 | 0.97 | 725.14 |
| Synonymous | SBS10a | 0.131783644 | 0.61 | 132.23 |
| splice_site | SBS10a | 0.022534337 | 0.75 | 23.55 |
| Stop_gain | SBS10b | 0.038504069 | 0.89 | 25.34 |
| Missense | SBS10b | 0.709148718 | 1.00 | 503.74 |
| Synonymous | SBS10b | 0.235045765 | 1.09 | 144.63 |
| splice_site | SBS10b | 0.017301448 | 0.58 | 13.81 |
| Stop_gain | SBS11 | 0.01848922 | 0.43 | 17.44 |
| Missense | SBS11 | 0.640900018 | 0.90 | 596.28 |
| Synonymous | SBS11 | 0.297348035 | 1.37 | 276.08 |
| splice_site | SBS11 | 0.043262728 | 1.44 | 37.50 |
| Stop_gain | SBS12 | 0.011616198 | 0.27 | 11.08 |
| Missense | SBS12 | 0.667138813 | 0.94 | 578.39 |
| Synonymous | SBS12 | 0.2916854 | 1.35 | 261.62 |
| splice_site | SBS12 | 0.029559589 | 0.98 | 23.44 |
| Stop_gain | SBS13 | 0.10045723 | 2.33 | 100.13 |
| Missense | SBS13 | 0.810516829 | 1.14 | 833.28 |
| Synonymous | SBS13 | 0.072808309 | 0.34 | 68.95 |
| splice_site | SBS13 | 0.016217632 | 0.54 | 16.75 |
| Stop_gain | SBS14 | 0.053464501 | 1.24 | 54.16 |
| Missense | SBS14 | 0.703106594 | 0.99 | 652.74 |
| Synonymous | SBS14 | 0.194786323 | 0.90 | 177.05 |
| splice_site | SBS14 | 0.048642583 | 1.62 | 46.91 |
| Stop_gain | SBS15 | 0.041115906 | 0.95 | 36.68 |
| Missense | SBS15 | 0.651392405 | 0.92 | 509.89 |
| Synonymous | SBS15 | 0.288714885 | 1.33 | 209.17 |
| splice_site | SBS15 | 0.018776804 | 0.62 | 16.29 |
| Stop_gain | SBS16 | 0.026966028 | 0.62 | 23.71 |
| Missense | SBS16 | 0.69656828 | 0.98 | 610.07 |
| Synonymous | SBS16 | 0.256640352 | 1.19 | 220.23 |
| splice_site | SBS16 | 0.01982534 | 0.66 | 17.71 |
| Stop_gain | SBS17a | 0.007785361 | 0.18 | 7.72 |
| Missense | SBS17a | 0.626552803 | 0.88 | 622.03 |
| Synonymous | SBS17a | 0.340413651 | 1.57 | 346.22 |
| splice_site | SBS17a | 0.025248185 | 0.84 | 26.52 |
| Stop_gain | SBS17b | 0.004291134 | 0.10 | 3.96 |
| Missense | SBS17b | 0.826582484 | 1.16 | 838.71 |
| Synonymous | SBS17b | 0.151512482 | 0.70 | 151.52 |
| splice_site | SBS17b | 0.0176139 | 0.59 | 17.36 |
| Stop_gain | SBS18 | 0.086961291 | 2.01 | 83.88 |
| Missense | SBS18 | 0.707393244 | 1.00 | 666.15 |
| Synonymous | SBS18 | 0.182016869 | 0.84 | 163.36 |
| splice_site | SBS18 | 0.023628595 | 0.79 | 22.30 |
| Stop_gain | SBS19 | 0.045604014 | 1.06 | 47.88 |
| Missense | SBS19 | 0.580013217 | 0.82 | 570.52 |
| Synonymous | SBS19 | 0.330316611 | 1.53 | 326.00 |
| splice_site | SBS19 | 0.044066158 | 1.47 | 44.00 |
| Stop_gain | SBS2 | 0.065784871 | 1.52 | 66.22 |
| Missense | SBS2 | 0.681964935 | 0.96 | 682.51 |
| Synonymous | SBS2 | 0.238920095 | 1.10 | 240.76 |
| splice_site | SBS2 | 0.013330099 | 0.44 | 13.56 |
| Stop_gain | SBS20 | 0.041480236 | 0.96 | 39.12 |
| Missense | SBS20 | 0.656689923 | 0.92 | 628.33 |
| Synonymous | SBS20 | 0.256548265 | 1.19 | 243.28 |
| splice_site | SBS20 | 0.045281575 | 1.51 | 44.92 |
| Stop_gain | SBS21 | 0.006296506 | 0.15 | 5.66 |
| Missense | SBS21 | 0.679275975 | 0.96 | 533.01 |
| Synonymous | SBS21 | 0.269614237 | 1.25 | 219.24 |
| splice_site | SBS21 | 0.044813282 | 1.49 | 31.76 |
| Stop_gain | SBS22 | 0.08173171 | 1.89 | 67.98 |
| Missense | SBS22 | 0.620064298 | 0.87 | 567.32 |
| Synonymous | SBS22 | 0.257513065 | 1.19 | 238.08 |
| splice_site | SBS22 | 0.040690928 | 1.35 | 36.04 |
| Stop_gain | SBS23 | 0.034702353 | 0.80 | 35.13 |
| Missense | SBS23 | 0.611253079 | 0.86 | 582.43 |
| Synonymous | SBS23 | 0.312648279 | 1.45 | 297.29 |
| splice_site | SBS23 | 0.04139629 | 1.38 | 38.62 |
| Stop_gain | SBS24 | 0.053930984 | 1.25 | 48.68 |
| Missense | SBS24 | 0.723788905 | 1.02 | 652.75 |
| Synonymous | SBS24 | 0.198182475 | 0.92 | 168.57 |
| splice_site | SBS24 | 0.024097637 | 0.80 | 21.55 |
| Stop_gain | SBS25 | 0.047912939 | 1.11 | 42.98 |
| Missense | SBS25 | 0.680533747 | 0.96 | 617.06 |
| Synonymous | SBS25 | 0.236710366 | 1.09 | 217.20 |
| splice_site | SBS25 | 0.034842948 | 1.16 | 31.11 |
| Stop_gain | SBS26 | 0.005740624 | 0.13 | 5.07 |
| Missense | SBS26 | 0.656603415 | 0.92 | 578.50 |
| Synonymous | SBS26 | 0.302507688 | 1.40 | 276.79 |
| splice_site | SBS26 | 0.035148273 | 1.17 | 29.42 |
| Stop_gain | SBS28 | 0.020989731 | 0.49 | 17.56 |
| Missense | SBS28 | 0.867066345 | 1.22 | 1026.92 |
| Synonymous | SBS28 | 0.098274651 | 0.45 | 94.71 |
| splice_site | SBS28 | 0.013669273 | 0.45 | 13.64 |
| Stop_gain | SBS29 | 0.062584741 | 1.45 | 56.02 |
| Missense | SBS29 | 0.73056677 | 1.03 | 652.49 |
| Synonymous | SBS29 | 0.177863808 | 0.82 | 148.85 |
| splice_site | SBS29 | 0.028984681 | 0.96 | 25.75 |
| Stop_gain | SBS3 | 0.038968929 | 0.90 | 36.93 |
| Missense | SBS3 | 0.71722413 | 1.01 | 671.94 |
| Synonymous | SBS3 | 0.210651878 | 0.97 | 199.00 |
| splice_site | SBS3 | 0.033155062 | 1.10 | 29.87 |
| Stop_gain | SBS30 | 0.060388306 | 1.40 | 61.55 |
| Missense | SBS30 | 0.644359536 | 0.91 | 613.12 |
| Synonymous | SBS30 | 0.26365664 | 1.22 | 247.96 |
| splice_site | SBS30 | 0.031595518 | 1.05 | 28.11 |
| Stop_gain | SBS31 | 0.034569439 | 0.80 | 32.93 |
| Missense | SBS31 | 0.651065188 | 0.92 | 613.67 |
| Synonymous | SBS31 | 0.272963889 | 1.26 | 260.09 |
| splice_site | SBS31 | 0.041401484 | 1.38 | 39.07 |
| Stop_gain | SBS32 | 0.038820182 | 0.90 | 35.44 |
| Missense | SBS32 | 0.643273916 | 0.91 | 548.07 |
| Synonymous | SBS32 | 0.27585603 | 1.27 | 234.00 |
| splice_site | SBS32 | 0.042049873 | 1.40 | 34.43 |
| Stop_gain | SBS33 | 0.018325667 | 0.42 | 17.37 |
| Missense | SBS33 | 0.631926463 | 0.89 | 583.61 |
| Synonymous | SBS33 | 0.324673519 | 1.50 | 298.47 |
| splice_site | SBS33 | 0.025074351 | 0.83 | 22.49 |
| Stop_gain | SBS34 | 0.130923564 | 3.03 | 90.39 |
| Missense | SBS34 | 0.682706698 | 0.96 | 577.44 |
| Synonymous | SBS34 | 0.170067309 | 0.79 | 124.81 |
| splice_site | SBS34 | 0.01630243 | 0.54 | 12.24 |
| Stop_gain | SBS35 | 0.037298089 | 0.86 | 35.17 |
| Missense | SBS35 | 0.700524366 | 0.99 | 673.35 |
| Synonymous | SBS35 | 0.216191461 | 1.00 | 210.92 |
| splice_site | SBS35 | 0.045986084 | 1.53 | 43.15 |
| Stop_gain | SBS36 | 0.121289746 | 2.81 | 121.31 |
| Missense | SBS36 | 0.68397378 | 0.96 | 667.94 |
| Synonymous | SBS36 | 0.169408487 | 0.78 | 159.95 |
| splice_site | SBS36 | 0.025327987 | 0.84 | 24.77 |
| Stop_gain | SBS37 | 0.017123856 | 0.40 | 13.90 |
| Missense | SBS37 | 0.720148088 | 1.01 | 628.04 |
| Synonymous | SBS37 | 0.236285454 | 1.09 | 206.60 |
| splice_site | SBS37 | 0.026442602 | 0.88 | 20.17 |
| Stop_gain | SBS38 | 0.051442348 | 1.19 | 53.60 |
| Missense | SBS38 | 0.671392395 | 0.95 | 685.59 |
| Synonymous | SBS38 | 0.239865345 | 1.11 | 240.21 |
| splice_site | SBS38 | 0.037299912 | 1.24 | 37.64 |
| Stop_gain | SBS39 | 0.035276308 | 0.82 | 31.47 |
| Missense | SBS39 | 0.756903654 | 1.07 | 687.84 |
| Synonymous | SBS39 | 0.178218615 | 0.82 | 158.51 |
| splice_site | SBS39 | 0.029601424 | 0.98 | 26.40 |
| Stop_gain | SBS4 | 0.066910025 | 1.55 | 62.44 |
| Missense | SBS4 | 0.690060638 | 0.97 | 645.38 |
| Synonymous | SBS4 | 0.208177983 | 0.96 | 194.69 |
| splice_site | SBS4 | 0.034851354 | 1.16 | 32.67 |
| Stop_gain | SBS40 | 0.052068888 | 1.21 | 50.52 |
| Missense | SBS40 | 0.720897975 | 1.01 | 689.14 |
| Synonymous | SBS40 | 0.199101732 | 0.92 | 188.19 |
| splice_site | SBS40 | 0.027931405 | 0.93 | 25.43 |
| Stop_gain | SBS41 | 0.064512577 | 1.49 | 51.73 |
| Missense | SBS41 | 0.72722383 | 1.02 | 715.37 |
| Synonymous | SBS41 | 0.191453469 | 0.88 | 169.90 |
| splice_site | SBS41 | 0.016810124 | 0.56 | 15.07 |
| Stop_gain | SBS42 | 0.04583875 | 1.06 | 43.60 |
| Missense | SBS42 | 0.668913724 | 0.94 | 630.60 |
| Synonymous | SBS42 | 0.250497382 | 1.16 | 236.42 |
| splice_site | SBS42 | 0.034750144 | 1.16 | 31.25 |
| Stop_gain | SBS44 | 0.043318474 | 1.00 | 40.89 |
| Missense | SBS44 | 0.656791028 | 0.92 | 609.60 |
| Synonymous | SBS44 | 0.268679024 | 1.24 | 245.96 |
| splice_site | SBS44 | 0.031211475 | 1.04 | 29.06 |
| Stop_gain | SBS5 | 0.036948015 | 0.86 | 34.10 |
| Missense | SBS5 | 0.686425929 | 0.97 | 620.62 |
| Synonymous | SBS5 | 0.248106813 | 1.15 | 222.56 |
| splice_site | SBS5 | 0.028519244 | 0.95 | 25.27 |
| Stop_gain | SBS6 | 0.042995149 | 1.00 | 34.45 |
| Missense | SBS6 | 0.628323289 | 0.88 | 452.49 |
| Synonymous | SBS6 | 0.310457684 | 1.43 | 211.26 |
| splice_site | SBS6 | 0.018223879 | 0.61 | 14.66 |
| Stop_gain | SBS7a | 0.047168161 | 1.09 | 46.77 |
| Missense | SBS7a | 0.673606897 | 0.95 | 629.42 |
| Synonymous | SBS7a | 0.25499179 | 1.18 | 239.73 |
| splice_site | SBS7a | 0.024233152 | 0.81 | 23.11 |
| Stop_gain | SBS7b | 0.036594038 | 0.85 | 36.69 |
| Missense | SBS7b | 0.636342434 | 0.90 | 592.20 |
| Synonymous | SBS7b | 0.29157009 | 1.35 | 276.33 |
| splice_site | SBS7b | 0.035493438 | 1.18 | 33.93 |
| Stop_gain | SBS7c | 0.062570181 | 1.45 | 71.92 |
| Missense | SBS7c | 0.732607843 | 1.03 | 813.63 |
| Synonymous | SBS7c | 0.187128568 | 0.86 | 196.84 |
| splice_site | SBS7c | 0.017693408 | 0.59 | 16.96 |
| Stop_gain | SBS7d | 0.028659463 | 0.66 | 28.24 |
| Missense | SBS7d | 0.660418342 | 0.93 | 576.03 |
| Synonymous | SBS7d | 0.293388919 | 1.36 | 254.28 |
| splice_site | SBS7d | 0.017533276 | 0.58 | 14.58 |
| Stop_gain | SBS8 | 0.059245473 | 1.37 | 57.25 |
| Missense | SBS8 | 0.724483527 | 1.02 | 672.36 |
| Synonymous | SBS8 | 0.182802847 | 0.84 | 172.23 |
| splice_site | SBS8 | 0.033468154 | 1.11 | 29.67 |
| Stop_gain | SBS84 | 0.035332354 | 0.82 | 33.41 |
| Missense | SBS84 | 0.676043217 | 0.95 | 636.15 |
| Synonymous | SBS84 | 0.258673465 | 1.20 | 243.00 |
| splice_site | SBS84 | 0.02995104 | 1.00 | 27.12 |
| Stop_gain | SBS85 | 0.048654549 | 1.13 | 39.89 |
| Missense | SBS85 | 0.717357803 | 1.01 | 659.81 |
| Synonymous | SBS85 | 0.213933928 | 0.99 | 198.52 |
| splice_site | SBS85 | 0.020053689 | 0.67 | 15.73 |
| Stop_gain | SBS86 | 0.032613404 | 0.76 | 28.79 |
| Missense | SBS86 | 0.800086573 | 1.13 | 733.26 |
| Synonymous | SBS86 | 0.134130123 | 0.62 | 116.58 |
| splice_site | SBS86 | 0.03316966 | 1.10 | 28.81 |
| Stop_gain | SBS87 | 0.037038355 | 0.86 | 23.89 |
| Missense | SBS87 | 0.664905033 | 0.94 | 426.60 |
| Synonymous | SBS87 | 0.277174571 | 1.28 | 161.49 |
| splice_site | SBS87 | 0.020887471 | 0.69 | 14.61 |
| Stop_gain | SBS88 | 0.033351782 | 0.77 | 29.81 |
| Missense | SBS88 | 0.745646931 | 1.05 | 735.90 |
| Synonymous | SBS88 | 0.208250242 | 0.96 | 193.29 |
| splice_site | SBS88 | 0.012751041 | 0.42 | 11.44 |
| Stop_gain | SBS89 | 0.048634194 | 1.13 | 44.95 |
| Missense | SBS89 | 0.699744639 | 0.98 | 638.75 |
| Synonymous | SBS89 | 0.217094867 | 1.00 | 198.16 |
| splice_site | SBS89 | 0.034564218 | 1.15 | 30.59 |
| Stop_gain | SBS9 | 0.051339574 | 1.19 | 42.77 |
| Missense | SBS9 | 0.707674677 | 1.00 | 661.87 |
| Synonymous | SBS9 | 0.217025348 | 1.00 | 196.55 |
| splice_site | SBS9 | 0.0239604 | 0.80 | 20.62 |
| Stop_gain | SBS90 | 0.167759836 | 3.89 | 119.09 |
| Missense | SBS90 | 0.596668422 | 0.84 | 529.14 |
| Synonymous | SBS90 | 0.211727272 | 0.98 | 149.36 |
| splice_site | SBS90 | 0.023818182 | 0.79 | 15.47 |

**Table S3: The genes used for the signature potential damage analysis**

| Gene symbol | Gene id |
| --- | --- |
| TP53 | 7157 |
| KRAS | 3845 |
| NRAS | 4893 |
| BRAF | 673 |
| BRCA2 | 675 |
| CDKN2A | 1029 |
| ARID1A | 8289 |
| PTEN | 5728 |
| TERT | 7015 |

**Table S4: Overview of the source of the replication timing data**

| B lympocyte line File type Data type |
| --- |
| GM06990 bigWig Wavelet-smoothed Signal |
| GM12801 bigWig Wavelet-smoothed Signal |
| GM12812 bigWig Wavelet-smoothed Signal |
| GM12813 bigWig Wavelet-smoothed Signal |
| GM12878 bigWig Wavelet-smoothed Signal |
| Files can be downloaded here: http://hgdownload.cse.ucsc.edu/goldenpath/hg19/encodeDCC/wgEncodeUwRepliSeq/ |
